# Supplementary material for: Regulation of antitumour CD8 T-cell immunity and checkpoint blockade immunotherapy by Neuropilin-1
Source: Nat Commun. 2019 Jul 26;10:3345. doi: 10.1038/s41467-019-11280-z (PMC6659631; doi:10.1038/s41467-019-11280-z)
Supplement: Supplementary file 1 — Supplementary Information [file 41467_2019_11280_MOESM1_ESM.pdf]

Supplementary Information

Regulation of anti-tumor CD8 T-cell immunity and checkpoint blockade immunotherapy by Neuropilin-1

Leclerc M et al.,

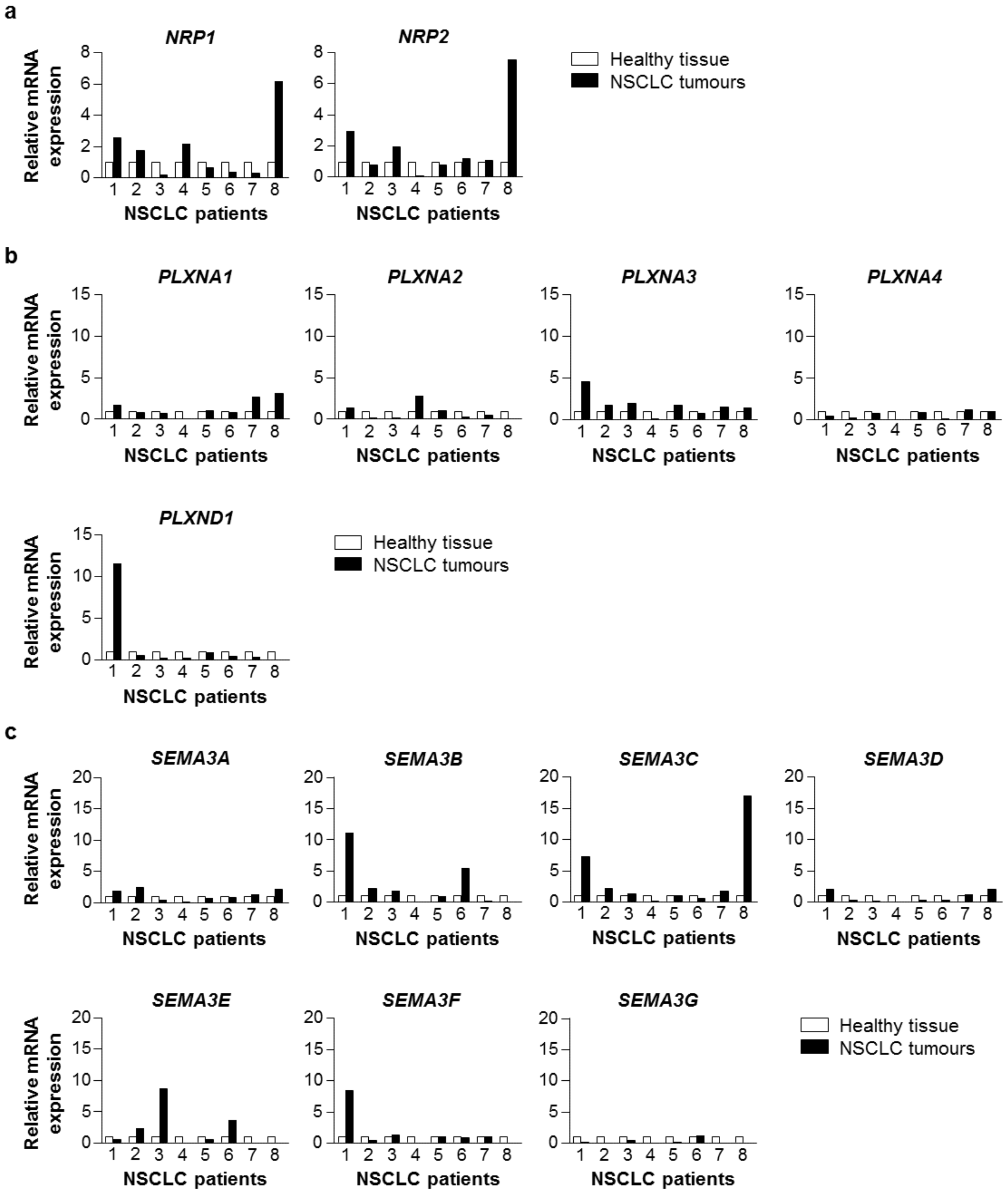

Supplementary Figure 1: Expression of *NRP*, *PLXN* and *SEMA3* genes in human lung tumour samples and autologous healthy lung tissues. **a**. Relative expression of *NRP1* and *NRP2* transcripts in fresh NSCLC tumours performed by qRT-PCR analysis. **b**. Relative expression of *PLXNA1*, *PLXNA2*, *PLXNA3*, *PLXNA4* and *PLXND1* transcripts in NSCLC tumours performed by qRT-PCR analysis. **c**. Relative expression of *SEMA3A*, *SEMA3B*, *SEMA3C*, *SEMA3D*, *SEMA3E*, *SEMA3F* and *SEMA3G* transcripts in fresh human lung tumours. Expression was normalized to autologous healthy lung tissues (n=8). Sequences of primer pairs are provided in Supplementary Table 3.

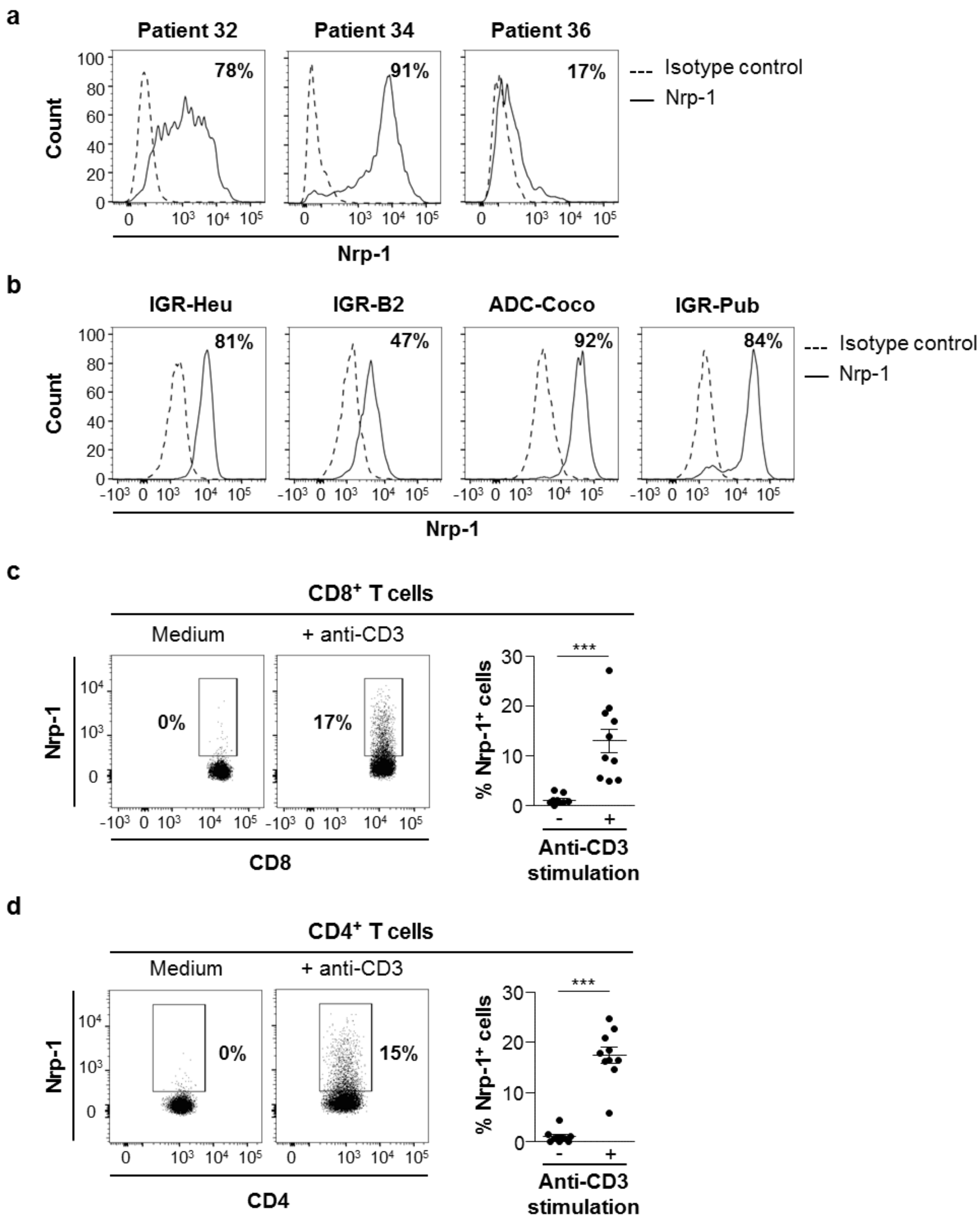

Supplementary Figure 2: Expression of Nrp-1 in human cells. **a.** Surface expression of Nrp-1 on freshly isolated NSCLC tumours cells was determined by immunofluorescence analysis. Percentages of positive cells are indicated. Full line: anti-Nrp-1; dashed line: isotypic control mAb. **b.** Surface expression of Nrp-1 in human NSCLC tumour cell lines was determined by immunofluorescence analysis. Percentages of positive cells are indicated. Full line: anti-Nrp-1; dashed line: isotypic control mAb. **c.** Expression of Nrp-1 on $CD8^+$ T lymphocytes from healthy donor PBL unstimulated (medium) and stimulated with immobilized anti-CD3 mAb (n=10). **d.** Expression of Nrp-1 on $CD4^+$ T lymphocytes from healthy donor PBL unstimulated (medium) and stimulated with immobilized anti-CD3 (n=10). Means ± SEM two-tailed Student's paired t test (**c**, **d**). *** $P \leq 0.001$.

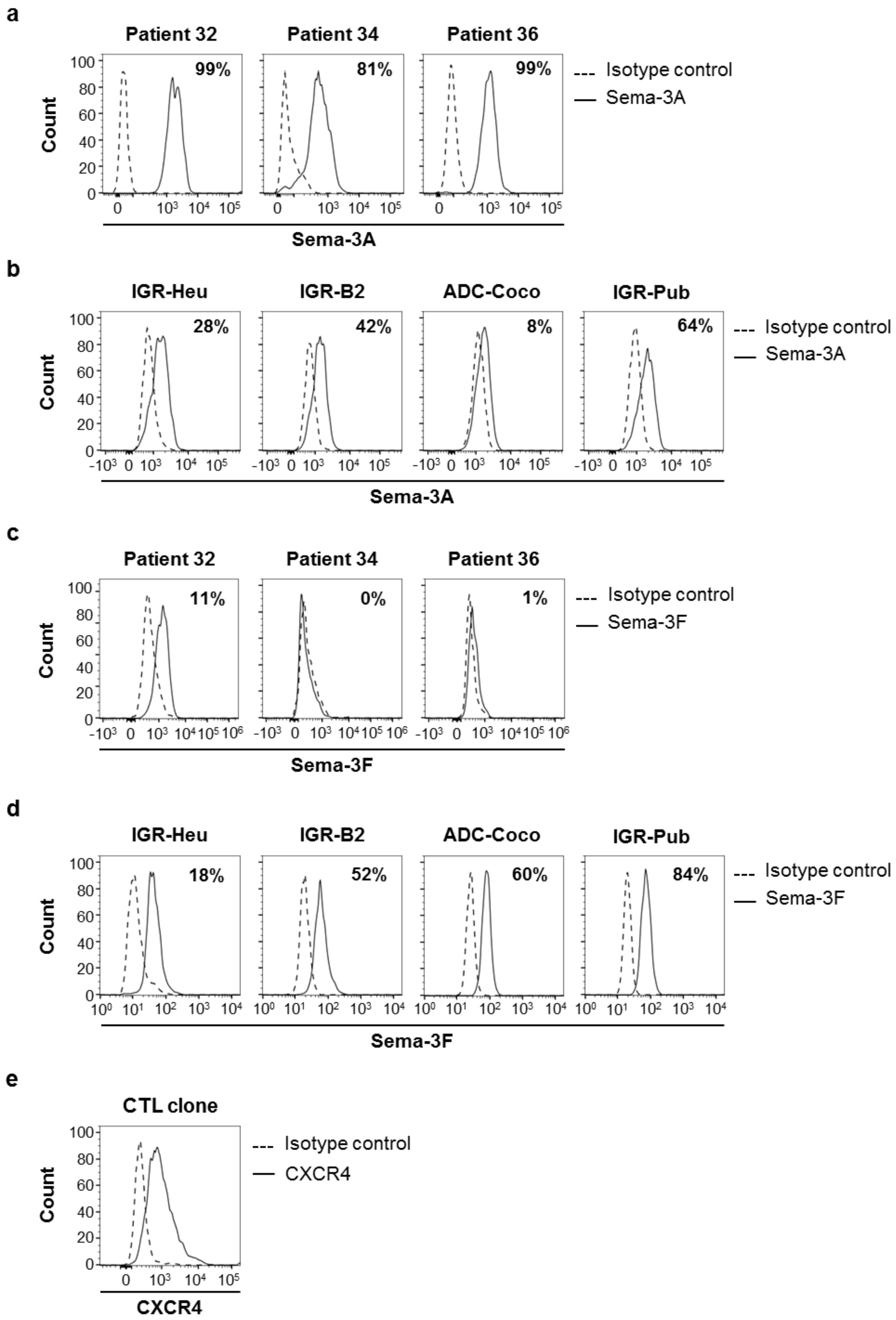

Supplementary Figure 3: Expression of Sema-3A, Sema-3F and CXCR4 in human cells. **a.** Intracellular expression of Sema-3A in freshly isolated human NSCLC tumours cells was determined by intracellular immunofluorescence analysis. Percentages of positive cells are indicated. Full line: anti-Sema-3A; dashed line: isotypic control. **b.** Intracellular expression of Sema-3A in NSCLC tumour cell lines was determined by immunofluorescence. Percentages of positive cells are indicated. Full line: anti-Sema-3A; dashed line: isotypic control. **c.** Intracellular expression of Sema-3F in freshly isolated human NSCLC tumours cells was determined by intracellular immunofluorescence analysis. Percentages of positive cells are indicated. Full line: anti-Sema-3F; dashed line: isotypic control. **d.** Intracellular expression of Sema-3F in NSCLC tumour cell lines was determined by immunofluorescence. Percentages of positive cells are indicated. Full line: anti-Sema-3F; dashed line: isotypic control. **e.** Expression of CXCR4 chemokine receptor on the human P62 CTL clone surface. Full line: anti-CXCR4 mAb; dashed line: isotypic control.

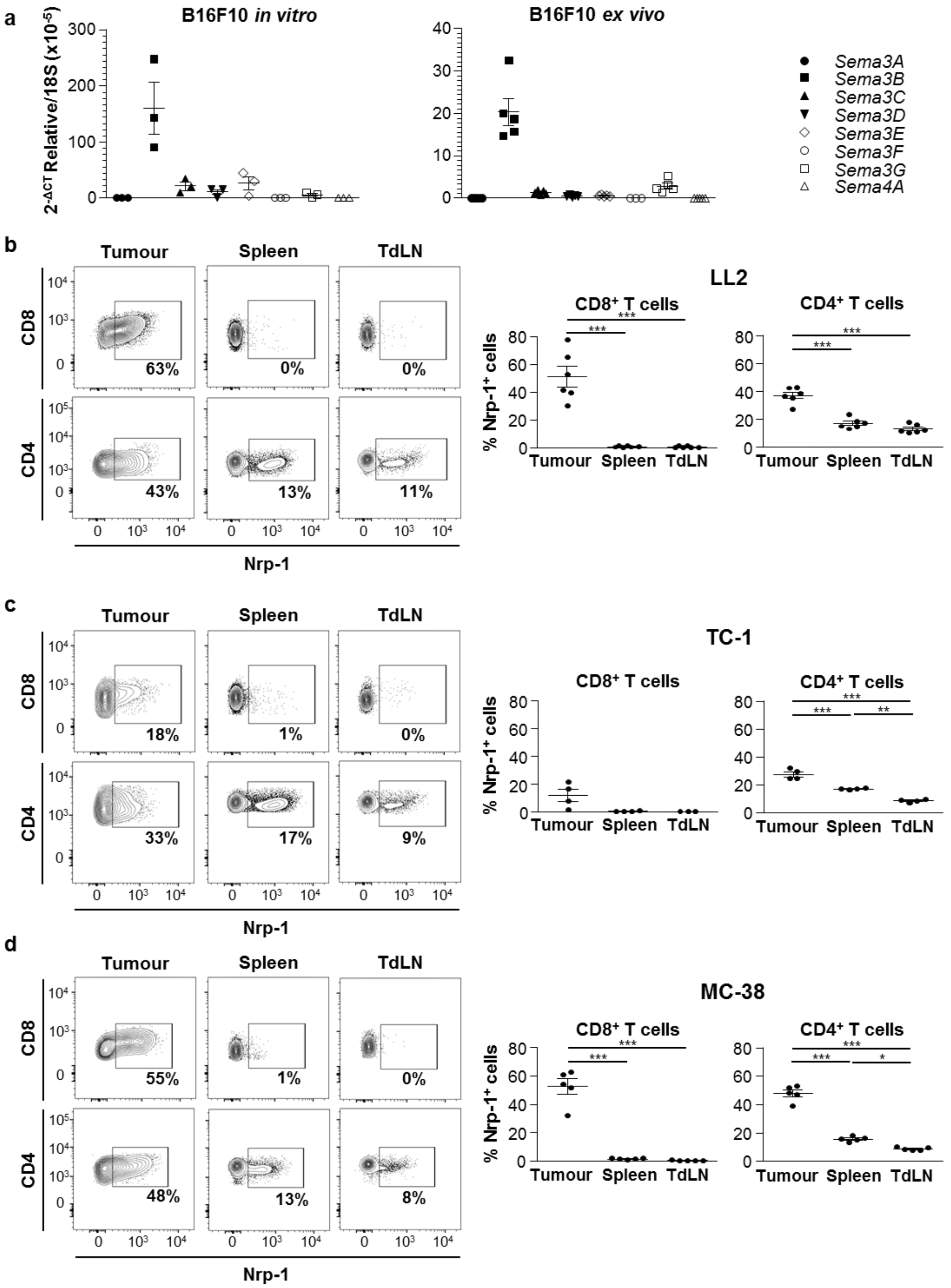

Supplementary Figure 4: Expression of Sema-3B and Nrp-1 in murine tumour and T cells. **a.** Relative expression of *Sema3A, Sema3B, Sema3C, Sema3D, Sema3E, Sema3F, Sema3G* and *Sema4A* transcripts in B16F10 tumour cells cultured *in vitro* (left) or engrafted in C57BL/6 mice (right) was determined by qRT-PCR analysis. **b**. Surface expression of Nrp-1 on CD4$^+$ and CD8$^+$ T cells infiltrating LL2 lung tumour cells engrafted in C57BL/6 mice at day 15. T lymphocytes from spleens and TdLN of tumour-bearing mice were analysed in parallel. Right: percentages of Nrp-1$^+$ cells among CD8$^+$ and CD4$^+$ T cells in TIL, splenocytes and TdLN (n=6). **c**. Surface expression of Nrp-1 on CD4$^+$ and CD8$^+$ T cells infiltrating TC-1 lung tumour cells engrafted in C57BL/6 mice at day 15. T lymphocytes from spleens and TdLN of tumour-bearing mice were analysed in parallel. Right: percentages of Nrp-1$^+$ cells among CD8$^+$ and CD4$^+$ T cells in TIL, splenocytes and TdLN (n=4). **d**. Surface expression of Nrp-1 on CD4$^+$ and CD8$^+$ T cells infiltrating MC-38 colon tumour cells engrafted in C57BL/6 mice at day 15. T lymphocytes from spleens and TdLN of tumour-bearing mice were analysed in parallel. Right: percentages of Nrp-1$^+$ cells among CD8$^+$ and CD4$^+$ T cells in TIL, splenocytes and TdLN (n=5). Means ± SEM one-way ANOVA test with Bonferroni correction (**b**, **c**, **d**). * $P \leq 0.05$; ** $P \leq 0.01$; *** $P \leq 0.001$. Sequences of primer pairs are provided in Supplementary Table 3.

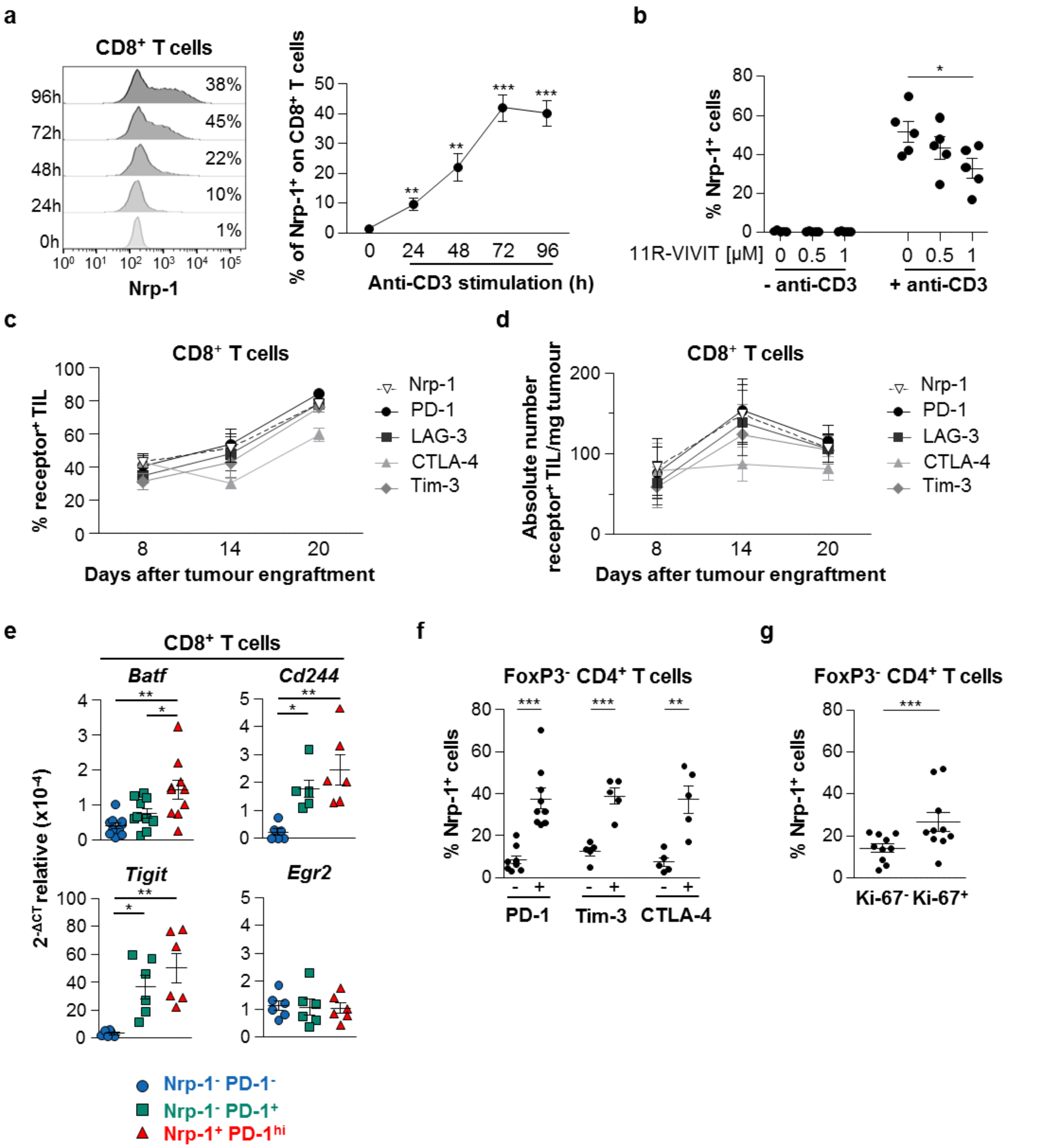

Supplementary Figure 5: Expression of Nrp-1 and transcription factors in TIL from the B16F10 melanoma model. **a.** Kinetic studies of Nrp-1 expression on $CD8^+$ T cells from naive mouse splenocytes unstimulated or stimulated with immobilized anti-CD3 mAb. **b.** The NFAT inhibitor 11R-VIVIT inhibits expression of Nrp-1 induced by anti-CD3 activation on $CD8^+$ T cells from naive mouse splenocytes in a dose-dependent manner. **c.** Kinetic studies of Nrp-1, PD-1, LAG-3, CTLA-4 and Tim-3 induction on $CD8^+$ TIL from B16F10 melanoma engrafted in C57BL/6 mice. Percentages of inhibitory receptor-expressing $CD8^+$ T lymphocytes are shown. **d.** Absolute numbers of $CD8^+$ TIL expressing Nrp-1, PD-1, LAG-3, CTLA-4 or Tim-3 per milligram of tumour. **e.** Relative expression of *Batf*, *Cd244*, *Tigit* and *Egr2* transcripts in Nrp-1^+PD-1^{hi}, Nrp-1^-PD-1^+ and Nrp-1^-PD-1^- $CD8^+$ T-cell subsets from B16F10 TIL was determined by qRT-PCR analysis at day 15 after tumour engraftment. **f.** Percentages of Nrp-1^+ on $Foxp3^-$ $CD4^+$ T lymphocytes from B16F10 expressing or not PD-1 (n=9), Tim-3 (n=5) or CTLA-4 (n=5). **g.** Percentages of Nrp-1^+ on $Foxp3^-$ $CD4^+$ T lymphocytes infiltrating B16F10 melanoma expressing or not Ki-67(n=10). Means ± SEM one-way ANOVA test with Bonferroni correction (**a**, **b**, **e**) or two-tailed Student's unpaired t test (**f**, **g**). * $P < 0.05$; ** $P < 0.01$; *** $P < 0.001$. Primer pairs are provided in Supplementary Table 3.

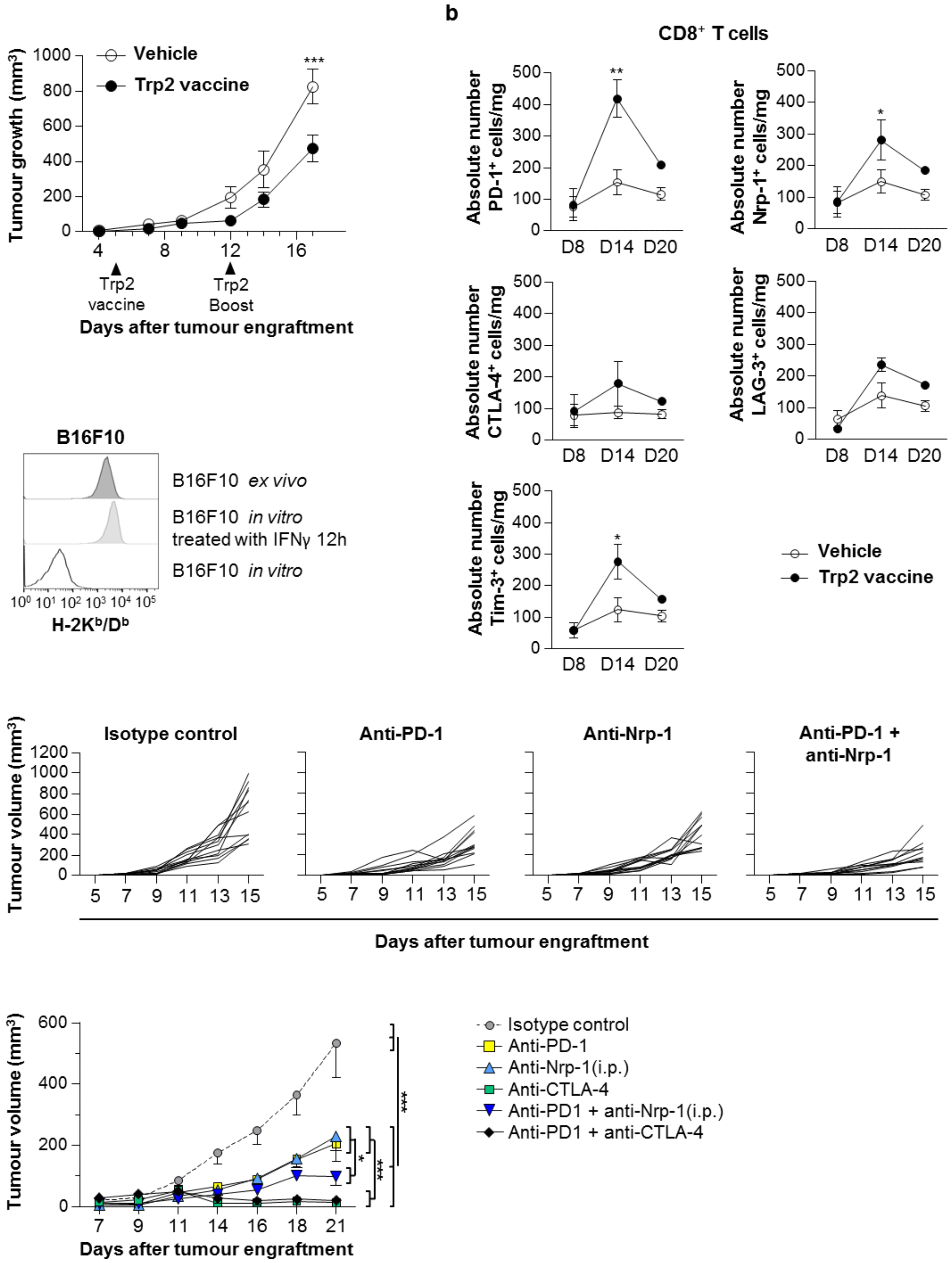

Supplementary Figure 6: Growth and infiltration of murine tumours engrafted in C57BL/6 mice. **a.** Mice were engrafted with B16F10 cells and then vaccinated with Trp2 peptide delivered with poly(I:C) at indicated time points (arrows) or with poly(I:C) control alone (vehicle). Tumour volumes are given as means (± SEM) of 5 mice/group. **b.** Kinetic studies of inhibitory receptor expression on $CD8^+$ T cells infiltrating B16F10. Absolute numbers of $CD8^+$ T cells expressing inhibitory receptors in TIL from Trp-2-vaccinated mice and control mice. Numbers of PD-1^+, Nrp-1^+, LAG-3^+, CTLA-4^+ and Tim-3^+ $CD8^+$ TIL per milligram of tumour are determined. **c.** Increase in MHC-I expression on B16F10 cells cultured in the presence of IFNγ or engrafted in C57BL/6 mice. H2-K^b/-D^b expression profiles of B16F10 cells cultured *in vitro* in medium alone or with IFNγ for 12 h, or isolated *ex vivo* at day 15. **d.** Growth of B16F10 tumours engrafted in C57BL/6 mice treated with blocking anti-Nrp-1, -PD-1, a combination of both mAb or an isotype control. Individual mouse tumours are shown. **e.** C57BL/6 mice were engrafted with MC-38 tumour cells and then treated i.p. with anti-PD-1, anti-Nrp-1 or anti-CTLA-4, or a combination of anti-PD-1 plus anti-Nrp-1 or anti-PD-1 plus anti-CTLA-4 mAb injected at days 4, 7, 10 and 16 after tumour inoculation. Tumour volumes are given as means (± SEM) of 5 mice/group. Means ± SEM two-way ANOVA test with Bonferroni correction (**a**, **b**, **e**). * $P < 0.05$; ** $P < 0.01$; *** $P < 0.001$.

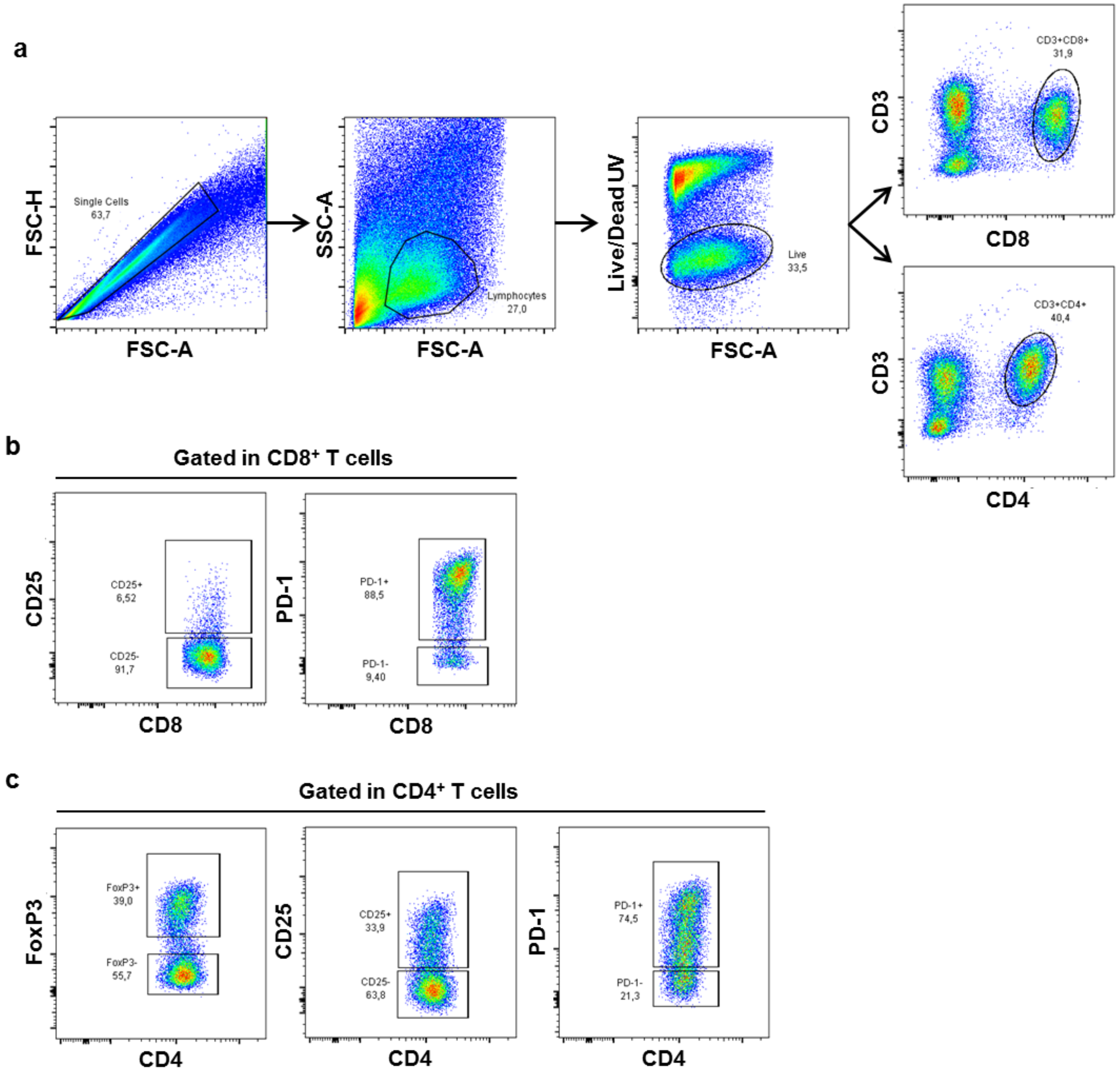

Supplementary Figure 7: Gating strategy for human T cells. Representative flow cytometry plot illustrating gating strategy identifying $CD3^{+}CD8^{+}$ cells and $CD3^{+}CD4^{+}$ cells from NSCLC tumour (**a**). Representative flow cytometry plot illustrating gating for CD25 and PD-1 in $CD8^{+}$ T cells from NSCLC tumour (**b**). Representative flow cytometry plot illustrating gating for FoxP3, CD25 and PD-1 in $CD4^{+}$ T cells from NSCLC tumour (**c**).

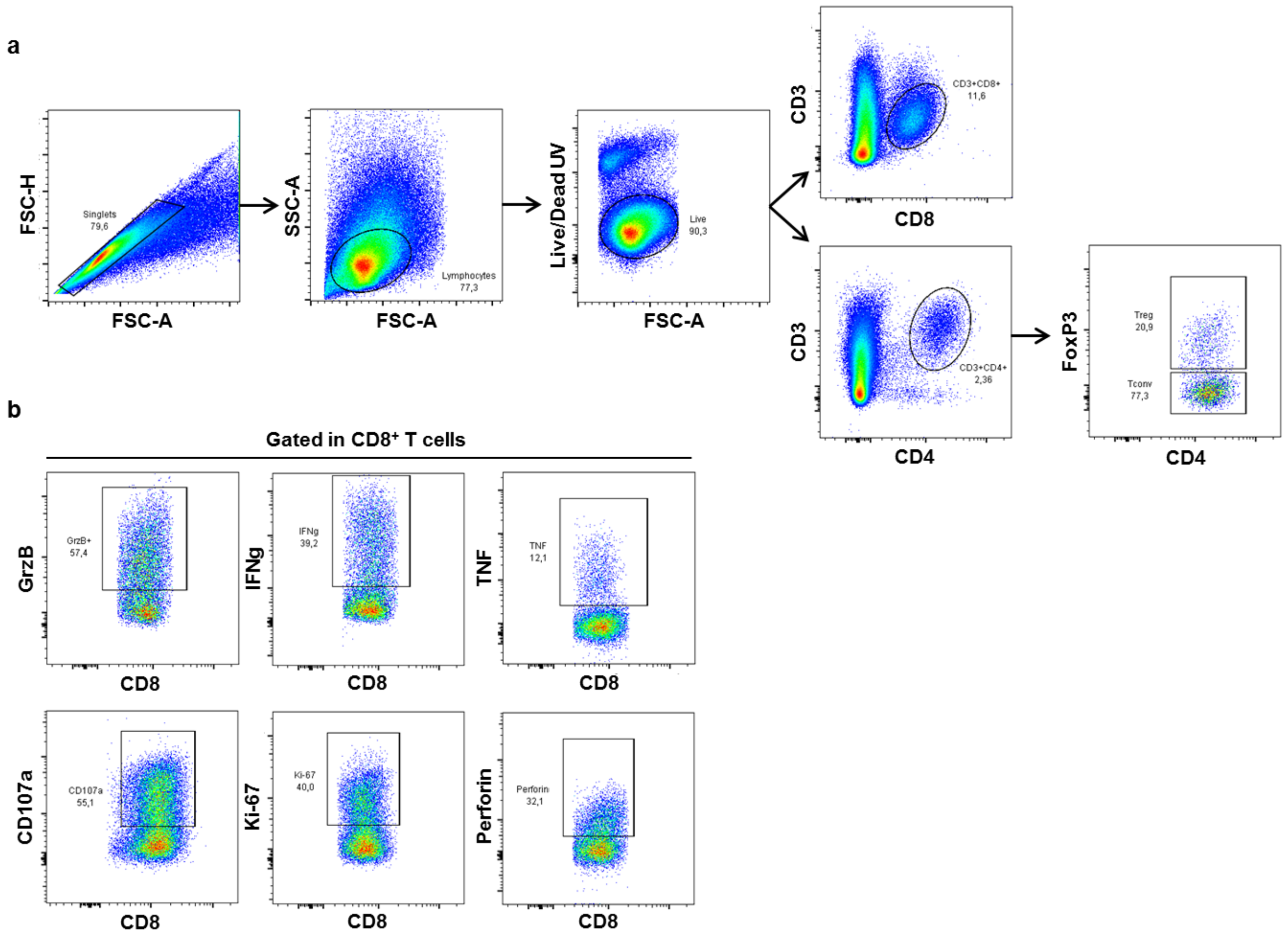

Supplementary Figure 8: Gating strategy for mouse T cells. Representative flow cytometry plot illustrating gating strategy identifying $CD3^{+}$ $CD8^{+}$ cells and $CD3^{+}$ $CD4^{+}$ cells ($FoxP3^{-}$ and $FoxP3^{+}$) from mouse tumour, spleen and TdLN (**a**). Representative flow cytometry plot illustrating gating for GrzB, IFNγ, TNF, CD107a, Ki-67 and Perforin in $CD8^{+}$ T cells from mouse tumour (**b**).

Supplementary Table 1: Absence of correlation between Nrp-1 and FoxP3 expression in $CD4^+$ TIL

	**% Nrp-1**	
**Patient**	**$CD3^+CD4^+FoxP3^-$**	**$CD3^+CD4^+FoxP3^+$**
**1**	12.20	9.64
**2**	10.70	9.87
**3**	1.38	31.50
**4**	7.28	14.00
**5**	14.00	16.60
**6**	9.65	9.14
**7**	21.20	10.70
**8**	6.27	8.56
**9**	8.17	12.80
**MEAN**	**10.09**	**13.65**
**SEM**	**3.94**	**4.70**

Supplementary Table 2: B16F10 tumour growth in treated mice

	Treatment			
**Day**	**Isotype**	**Anti-PD-1**	**Anti-Nrp-1**	**Anti-PD-1 + anti-Nrp-1**
**7**	12.04 ± 1.66	12.71 ± 4.34	10.33 ± 2.06	8.49 ± 2.70
**9**	46.10 ± 9.34	44.34 ± 18.64	44.00 ± 9.50	19.76 ± 5.63
**11**	167.77 ± 23.51	99.99 ± 23.96	111.47 ± 23.45	68.74 ± 15.24
**13**	299.72 ± 53.88	159.44 ± 24.18	199.60 ± 27.74	116.50 ± 21.49
**15**	575.06 ± 122.19	321.55 ± 53.43	410.45 ± 67.29	213.22 ± 44.58

Mean values of tumour volumes are in mm^3 ± SEM

Supplementary Table 3: List of human and mouse primer pairs used

Human Gene	Primer1 (Forward) 5' - 3'	Primer2 (Reverse) 5' - 3'
NRP1	CAAGACTTACAAGATCGACG	AGAACAGGTTTGTTTCCTTC
NRP2	AGGTATTTCAAGCCAACAAC	CGGATTCTAACAAACCTTGTC
PLXNA1	AGAGGTACTATGCAGACATC	TGTACTTGGTGATGTAGGAG
PLXNA2	CTGATCTATCTGCGGGTATC	CTGACAAATATCTTCCCTGTC
PLXNA3	GAGGTATTATCGAGACATTGC	CTTGGTGACATAGAAATACAGC
PLXNA4	GAAAACATGATCCGGTACAC	AGGTAGATTTCAGACACCATC
PLXND1	GTGACTATGGGAACAACATC	AGTCGTAGATGGTGAAATTG
SEMA3A	GATCCAAAGTTCATTAGTGCC	GCATATCTGACCTATTCTAGC
SEMA3B	AGGACATTGGTACTGAGTG	CATCCTCTATCCTTCCTGG
SEMA3C	ATCAATGAGGAGCTTTTCTC	TTCCTCTTGGTTAAACTTCG
SEMA3D	GAACTGTCCTCAAAGTTGTC	TGCTTGAATATCTGCAACTC
SEMA3E	GCAGAGAATACTGGTGAATAAG	CACTGGATTCTTATGATCTCTG
SEMA3F	CTTTACTTCTTCTTCCGTGAG	ATCAAAGTGAGTCTCAATGC
SEMA3G	CAAGGTGTACTTCTTCTTCTC	AAAGTGCTCCATTTGTTCAC
18S	CGGACAGGATTGACAGATTG	CAAATCGCTCCACCAACT

Mouse Gene	Primer1 (Forward) 5' - 3'	Primer2 (Reverse) 5' - 3'
Sema3A	TGGGCTGGTTCACTGGGATTGC	CTGGAGCTGTTGGCCAAGCCAT
Sema3B	GCTGTCTTCTCCACCTCCAG	ACATGCCAGGTCTTGGGTAG
Sema3C	ATG GCC ACT CTT GCT CTA GG	CAT CTT GTC TTC GGC TCC TC
Sema3D	GGA AAA GCG ACA AGA GTT GC	TGA AAA TTT TGT TTT TCA AAC ACT G
Sema3E	GGG GCA GAT GTC CTT TTG A	AGT CCA GCA AAC AGC TCA TTC
Sema3F	GAA GGA GGA ACG CGG AAG	AGG CAG TGA CAA GCA TCG T
Sema3G	GAAGCCGAGATGCCCTTTAC	GTCTTTTCCCTTGCGGACAC
Sema4A	CATGTATCTGGGTACCTCCAC	GGACTGTCAGGACTTCTTTAATTA
Batf	GGCAAACAGGACTCATCTGATGATG	GGCAGCCCGGCCTCAGTTTACATG
Cd244	AATTGGACAGGCGTGTTTCT	TCTCCAGGGAAAGTCTGCTG
Egr2	CTACCCGGTGGAAGACCTC	AATGTTGATCATGCCATCTCC
Tigit	CCACAGCAGGCACGATAGATA	CATGCCACCCCAGGTCAAC
18S	CGGACAGGATTGACAGATTG	CAAATCGCTCCACCAACT
