# Supplementary material for: Regulation of antitumour CD8 T-cell immunity and checkpoint blockade immunotherapy by Neuropilin-1
Source: Nat Commun. 2019 Jul 26;10:3345. doi: 10.1038/s41467-019-11280-z (PMC6659631; doi:10.1038/s41467-019-11280-z)
Supplement: Supplementary file 3 — Reporting Summary [file 41467_2019_11280_MOESM3_ESM.pdf]

nature research

Corresponding author(s): MAMI-CHOUAIB Fathia

Last updated by author(s): Jun 21, 2019

Reporting Summary

Nature Research wishes to improve the reproducibility of the work that we publish. This form provides structure for consistency and transparency in reporting. For further information on Nature Research policies, see Authors & Referees and the Editorial Policy Checklist.

Statistics

For all statistical analyses, confirm that the following items are present in the figure legend, table legend, main text, or Methods section.

n/a	Confirmed	
☐	☒	The exact sample size (*n*) for each experimental group/condition, given as a discrete number and unit of measurement
☐	☒	A statement on whether measurements were taken from distinct samples or whether the same sample was measured repeatedly
☐	☒	The statistical test(s) used AND whether they are one- or two-sided<br>*Only common tests should be described solely by name; describe more complex techniques in the Methods section.*
☒	☐	A description of all covariates tested
☐	☒	A description of any assumptions or corrections, such as tests of normality and adjustment for multiple comparisons
☐	☒	A full description of the statistical parameters including central tendency (e.g. means) or other basic estimates (e.g. regression coefficient) AND variation (e.g. standard deviation) or associated estimates of uncertainty (e.g. confidence intervals)
☒	☐	For null hypothesis testing, the test statistic (e.g. *F*, *t*, *r*) with confidence intervals, effect sizes, degrees of freedom and *P* value noted<br>*Give P values as exact values whenever suitable.*
☒	☐	For Bayesian analysis, information on the choice of priors and Markov chain Monte Carlo settings
☒	☐	For hierarchical and complex designs, identification of the appropriate level for tests and full reporting of outcomes
☒	☐	Estimates of effect sizes (e.g. Cohen's *d*, Pearson's *r*), indicating how they were calculated

Our web collection on statistics for biologists contains articles on many of the points above.

Software and code

Policy information about availability of computer code

Data collection	Flow cytometry data were collected on BD Fortessa instruments with FACSDiva software (BD).<br>qRT-PCR data were collected with rtqPCR step-one Plus (Applied).
Data analysis	Flow cytometry data were analyzed on FlowJo v10 (TreeStar) and statistical analysis performed on Prism v5 (GraphPad).

For manuscripts utilizing custom algorithms or software that are central to the research but not yet described in published literature, software must be made available to editors/reviewers. We strongly encourage code deposition in a community repository (e.g. GitHub). See the Nature Research guidelines for submitting code & software for further information.

Data

Policy information about availability of data

All manuscripts must include a data availability statement. This statement should provide the following information, where applicable:

- Accession codes, unique identifiers, or web links for publicly available datasets
- A list of figures that have associated raw data
- A description of any restrictions on data availability

The authors state that all data generated during this study are included in the article and its supplementary information file, and are available from the corresponding author upon reasonable request.

Field-specific reporting

Please select the one below that is the best fit for your research. If you are not sure, read the appropriate sections before making your selection.

☒ Life sciences ☐ Behavioural & social sciences ☐ Ecological, evolutionary & environmental sciences

For a reference copy of the document with all sections, see nature.com/documents/nr-reporting-summary-flat.pdf

Life sciences study design

All studies must disclose on these points even when the disclosure is negative.

Sample size	No sample size calculation was performed. Animal sample size were determined using previous studies and guided by the 3R principle. Human samples sizes were driven by the availability of biopsies.
Data exclusions	No data were excluded from the analysis
Replication	Results were reliably reproduced for each experiment. This included independant replication: all experiments presented in this study were performed using at least 2 biological replicates.
Randomization	- Human material was obtained from random donors and patients after informed written consent.<br>- Mice were randomized after tumour inoculation to obtain homogeneous groups.
Blinding	Blinding was used during measurement of tumours in different treatment groups, and also during the analysis where each mouse was sorted by their numbers and not by the experimental group.

Reporting for specific materials, systems and methods

We require information from authors about some types of materials, experimental systems and methods used in many studies. Here, indicate whether each material, system or method listed is relevant to your study. If you are not sure if a list item applies to your research, read the appropriate section before selecting a response.

Materials & experimental systems

n/a	Involved in the study	
☐	☒	Antibodies
☐	☒	Eukaryotic cell lines
☒	☐	Palaeontology
☐	☒	Animals and other organisms
☐	☒	Human research participants
☒	☐	Clinical data

Methods

n/a	Involved in the study	
☒	☐	ChIP-seq
☐	☒	Flow cytometry
☒	☐	MRI-based neuroimaging

Antibodies

Antibodies used

-> For flow cytometry:
- Human: anti-CD3 (clone UCHT1, Biolegend 300424, 1/200), -CD4 (clone RPA-T4, Biolegend 300517, 1/200), -CD8α (clone RPA-T8, Biolegend 301023, 1/200), -CD25 (clone CD25-3G10, Invitrogen MHCD2501, 1/100), -FoxP3 (clone 259D, Biolegend 320213, 1/50), -PD-1 (clone J105, eBioscience 25-2799-42, 1/100) and -Nrp-1 (clone 12C2, Biolegend 354504, 1/25) mAb, and -human IgG Fc (clone HP6017, Biolegend 409306, 1/100).
- Mouse: CD3 (clone 17A2, Biolegend 100241, 1/100), CD4 (clone RM4-5, Biolegend 100536, 1/200), CD8α (clone REA601, Miltenyi 130-109-252, 1/20), CTLA-4 (clone UC10-4B9, Miltenyi 130-102-570, 1/20), PD-1 (clone 29F.1A12, Biolegend 135223, 1/100), Tim-3 (clone RMT3-23, Biolegend 119705, 1/100), LAG-3 (clone C9B7W, Biolegend 125211, 1/100), Nrp-1 (clone 3E12, Biolegend 145209, 1/20), T-bet (clone REA102, Miltenyi 130-119-783, 1/50), NFATc1 (clone 7A6, Biolegend 649603, 1/100), Blimp-1 (clone 5E7, Biolegend 150005, 1/100), Helios (clone 22F6, Biolegend 137210, 1/25), IRF-4 (clone REA201, Miltenyi Biotec 130-100-915, 1/20), FoxP3(clone FJK-16s, Thermo Fischer Scientific 25-5773-82, 1/200), Ki-67 (clone REA183, Miltenyi Biotec 130-120-418, 1/50), Perforin (clone eBioOMAK-D, eBioscience 17-9392-80, 1/50), granzyme B (clone GB11, Biolegend 515403, 1/50), IFNγ (clone XMG1.2, Biolegend 505808, 1/200), and TNFα (clone MP6-XT22, Biolegend 506321, 1/100).

-> For western blot :
anti-mouse Sema-3B mAb (clone 904201, R&D systems MAB5440, 1µg/ml), anti-human Sema-3A (clone 215803, R&D systems MAB1250, 1µg/ml), anti-β-actin-peroxidase (clone AC-15, Merck A3854, 1/50 000), secondary anti-mouse (Santa cruz Biotechnology sc 2031) or anti-rat (R&D systems HAF005) HRP-conjugated Ab.

-> For functional assay and in vivo experiments (mouse):
Neutralizing anti-Nrp-1 mAb (clone 761704, R&D systems MAB59941, 10µg/mL), anti-PD-1 (clone RMP1-14, Bio-X-Cell BE0146, 10µg/mL) or isotype control (clone 2A3, Bio-X-Cell BE0089, 10µg/mL).

Validation | All commercial antibodies were used in the system under study (assay and species) according to the profile of manufacturer.

Eukaryotic cell lines

Policy information about cell lines

Cell line source(s)

-> Human cell line:
NSCLC cell line IGR-Pub was derived from the tumour specimen of patient Pub adenocarcinoma in one of our laboratory.
Autologous CTL clone P62 was derived from TIL of the same patient.
The allogeneic NSCLC cell lines IGR-B2, IGR-Heu, ADC-Coco, ADC-Tor and ADC-Let were derived from tumour specimens in one of our laboratories.
16HBE14o- (16HBE) (Merck, SCC150).
--> Mouse cell line:
The B16F10 melanoma cell line (H-2b) and MC-38 colon tumour cell line were purchased from the American Type Culture Collection and Kerafast (ATCC, CRL-6475™ and Kerafast, ENH204-FP, respectively).

Authentication

We regularly authenticate our NSCLC cell lines by testing recognition by autologous CTL clones and HLA-A2 expression when applicable.

Mycoplasma contamination

All the cell lines are mycoplasma-free and were regularly tested for mycoplasma contamination.

Commonly misidentified lines
(See ICLAC register)

NA

Animals and other organisms

Policy information about studies involving animals; ARRIVE guidelines recommended for reporting animal research

Laboratory animals

Female C57BL/6J mice (6 weeks old) were purchased from Envigo.

Wild animals

Study did not involve wild animals

Field-collected samples

Study did not involve samples collected in the field

Ethics oversight

All animals were housed at Gustave Roussy's animal facility and treated in accordance with guidelines established by the institutional animal committee (CEEA n°26: 2015-041-1229 and 2018-056-16280).

Note that full information on the approval of the study protocol must also be provided in the manuscript.

Human research participants

Policy information about studies involving human research participants

Population characteristics

- 12 Healthy donor blood samples were collected from the French blood bank (Etablissement français du Sang (EFS); agreement number N°12/EFS/079)
- 11 Patient blood samples were collected from Gustave Roussy. All patients were suffering from advanced and inoperable NSCLC stage IIIB/IV. Immune monitoring in the blood of patients was approved by the Kremlin-Bicêtre Hospital Ethics Committee (n° 110-08; ID RCB: 2008-A01171-54), and Declaration of Helsinki protocols were followed.
- 28 Fresh NSCLC tumours were obtained from the Centre chirurgical Marie Lannelongue and the Institut mutualiste Montsouris.

Recruitment

Human material was obtained from random donors and patients after informed written consent. Healthy donors and NSCLC patients were not selected by age or sex.

Ethics oversight

Etablissement français du Sang (EFS); agreement number N°12/EFS/079
The Kremlin-Bicêtre Hospital Ethics Committee (n° 110-08; ID RCB: 2008-A01171-54)

Note that full information on the approval of the study protocol must also be provided in the manuscript.

Flow Cytometry

Plots

Confirm that:

- [ ] The axis labels state the marker and fluorochrome used (e.g. CD4-FITC).
- [x] The axis scales are clearly visible. Include numbers along axes only for bottom left plot of group (a 'group' is an analysis of identical markers).
- [x] All plots are contour plots with outliers or pseudocolor plots.
- [x] A numerical value for number of cells or percentage (with statistics) is provided.

Methodology

Sample preparation

-> For human experiments :
For freshly isolated TIL, human lung tumours were dissociated mechanically and enzymatically using a tumour dissociation kit (Miltenyi Biotec 130-095-929). Mononuclear cells were then isolated by a Ficoll-Hypaque gradient, and epithelial tumour cells were isolated using a tumour cell isolation kit (Miltenyi 130-108-339).
For PBL, mononuclear cells were then isolated by a Ficoll-Hypaque gradient.
Cell surface and intracellular staining of human cells was performed on single-cell suspensions. Dead cells were excluded using the Live/Dead Fixable Blue Dead Cell Stain Kit (Invitrogen 1866842). For intracellular staining, cells were fixed/permeabilized with the Foxp3 Staining Buffer Set according to the manufacturer's instructions (eBioscience 00-5523-00).

-> For mouse experiments:
Cells were isolated from spleen and draining lymph nodes by crushing on 100 µm cell strainers
cell strainer. Cells were run through cell strainer and red blood cells were lysed in Red blood lysis buffer (Miltenyi Biotec 130-094-183) for 5 minutes. The cell suspension was then washed in PBS 2% fetal calf serum (FCS) and passed through a 100µm cell strainer an additional time.
Tumours were harvested at days 8-to-21 and digested for 40 min at 37°C according to Tumour Dissociation Kit protocol (Miltenyi Biotec 130-096-730). Tumours were crushed on 100 µm cell strainers and washed twice with PBS 2% FCS. Single cell suspensions were enriched for CD45+ cells or CD8+ cells using the MultiMACS system (Miltenyi Biotec). Briefly, cells from tumour tissues were labelled with anti-CD45 or anti-CD8a microbeads (Miltenyi Biotec 130-052-301 or 130-117-044 respectively), and then purified using the POSSEL program on MultiMACS. The positive fraction was recovered for TIL analysis by flow cytometry or ex vivo assays.
Cell surface and intracellular staining of mouse cells was performed on single-cell suspensions. Dead cells were excluded using the Live/Dead Fixable Blue Dead Cell Stain Kit (Invitrogen 1866842). For intracellular staining, cells were fixed/permeabilized with the Foxp3 Staining Buffer Set according to the manufacturer's instructions (eBioscience 00-5523-00).

Instrument

BD Fortessa and MultiMACS system (Miltenyi Biotec)

Software

FlowJo v10 (TreeStar)

Cell population abundance

Purity of cell population isolated by FACS or MultiMACS was typically >90%.
CD8 T cells from tumours represent at least >5% of the CD45+ cells isolated (mouse experiment).

Gating strategy

Refer to Supplementary Method.

☒ Tick this box to confirm that a figure exemplifying the gating strategy is provided in the Supplementary Information.
